# The hidden effects of COVID-19 on HIV services in Zanzibar: country report

**DOI:** 10.1186/s12981-023-00570-8

**Published:** 2023-10-17

**Authors:** Mansour Maulid Mshenga, Farhat Jowhar Khalid, Shaaban Hassan Haji, Tatu Bilali Ali, Khadija Abbas Mohamed, Damian Jeremia Damian

**Affiliations:** Zanzibar Integrated HIV, Hepatitis, TB and Leprosy Programme, Ministry of Health, P.O.BOX 1300, Zanzibar, Tanzania

**Keywords:** COVID 19, HIV services, Hidden effects, Zanzibar

## Abstract

The COVID-19 pandemic has had a major effect on HIV-related healthcare services. Zanzibar has experienced several interruptions of HIV services in the areas of testing and counselling, prevention of mother-to-child transmission of HIV, key population, care and treatment services as well due to the hospital operating at a reduced capacity and the strict visit restrictions with health care allocations to COVID 19 pandemic. The community HIV initiatives, such as index testing and ARVs medicine refills, were used to mitigate the consequences of the epidemic and can be applied in future emergencies. This report tries to reveal COVID-19’s unnoticed consequences on HIV services in Zanzibar.

## Introduction

In early 2020, a novel coronavirus, SARS-CoV-2, was identified as the causative agent of an outbreak of viral pneumonia disease (COVID-19), which began at the end of December 2019 in Wuhan Province, China. On March 11, 2020, the World Health Organization (WHO) declared COVID-19 as a pandemic disease [1]. The first confirmed case of COVID-19 was reported in Tanzania in March, 2020 and by the month of April, 2020, almost the COVID-19 had been reported countrywide including Zanzibar [2]. The COVID-19 pandemic has had devastating consequences on global healthcare systems, extending far beyond the direct impacts of the virus. One area profoundly affected is HIV services, particularly in countries with already fragile healthcare systems, such as Zanzibar [3].

Zanzibar, a semiautonomous region in the United Republic of Tanzania, comprising two main islands, Unguja and Pemba, along with several sparsely populated islets, is directly responsible for its own health services. Zanzibar's HIV prevalence is less than 1% in the general population but is concentrated among key populations, with estimated rates of 12.1% among female sex workers (FSWs), 5.1% among people who inject drugs (PWID), and 5.0% among men who have sex with men (MSM) [4].

Before the pandemic, Zanzibar had made significant progress in its fight against HIV/AIDS, implementing various strategies to enhance access to testing, prevention, and treatment services [5]. However, the arrival of COVID-19 abruptly halted these efforts. Experiences from affected countries have demonstrated that the COVID-19 outbreak can disrupt healthcare systems, including the provision of vital HIV services to people living with HIV (PLHIV) [6].

## HIV services in Zanzibar in 2020

In the fight against ending the HIV epidemic, Zanzibar conducted routine HIV services encompassing key interventions, including HIV Testing and Counselling (HTC), Prevention of Mother-to-Child Transmission of HIV (PMTCT), Key Population Services (KP), as well as Care and Treatment Centers (CTC) through the Zanzibar Integrated HIV, Hepatitis, TB, and Leprosy Programme (ZIHHTLP), which operates under the Directorate of Preventive Services (DPS) and Health Education of the Ministry of Health Zanzibar. As of December 2020, Zanzibar had 7020 clients who received care in 14 Care and Treatment clinics across the counties, with the goal of achieving the 95–95–95 targets by 2030 [7]. However, the COVID-19 pandemic disrupted Zanzibar's HIV services, as seen in the following four key areas:

### HIV testing and counselling (HTC) services

Despite the COVID 19 pandemic and its associated challenges, more clients were tested between July and December 2020 than between January and June 2020. Additionally, the country's percentage of clients who tested positive increased noticeably from 0.6% of clients between January and June 2020 to 0.7% of clients between July and December 2020. (Table 1)

**Table 1:** Number & percentage of clients receiving HIV testing services in Zanzibar, 2020

Tested status	January–June 2020	July–December 2020
Total tested	110,886	120,284
Tested positive	755 (0.6%)	869 (0.7%)

### Prevention of mother to child transmission of HIV (PMTCT) services

In the period of July to December 2020 compared to the period of January to June 2020, PMTCT services saw a decline in the number of pregnant women who were aware of their positive HIV status and those who received ART to lower the risk of mother-to-child transmission of HIV, as well as a decline in the proportion of infants born to HIV-positive mothers who received an HIV virological test within 2 months. (Table 2)

**Table 2:** Percentage of clients receiving PMTCT services in Zanzibar, 2020

Indicator	Jan–June, 2020	July–Dec, 2020
Percentage of pregnant women with known HIV status	100%	99.6%
Percentage of pregnant women receiving ART	100%	91.7%
Percentage of infants tested within 2 months	74.3%	87.6%

### Key population services (KP)

In January–June 2020 compared to July–December 2020, there was a considerable decline in the number of key populations (MSM, FSW, and PWID) that were reached, tested, and found to be HIV-positive. (Table 3)

**Table 3:** Number & percentage of Key Populations receiving HIV services in Zanzibar, 2020

Indicator	Jan–June, 2020			July–Dec, 2020		
Client category	Reached	Tested	Positive	Reached	Tested	Positive
MSM	451	386	8 (85.6%)	1469	1083	25 (73.7%)
FSW	1703	1499	53 (88.0%)	2735	2500	79 (91.4%)
PWID	933	679	12 (72.8%)	2149	1933	16 (89.9%)
Total	2883	2768	73 (96.0%)	6353	5516	120 (86.8%)

### Care and treatment centers (CTC) services

HIV care and treatment in Zanzibar were significantly harmed by the COVID-19 outbreak. In comparison to the other quarters of 2020, the facilities reported a decrease in the number of new clients who were enrolled, got care, and underwent treatment in April–June 2020. (Table 4)

**Table 4:** Number of patients receiving HIV Care and Treatment services in Zanzibar, 2020

Indicators	Jan–Mar	Apr–June	July–Sept	Oct–Dec
Client on care	6830	6797	6889	7020
Client on treatment	6644	6604	6800	6940
New enrolled	305	211	246	257

## Discussion

Despite the presence of COVID-19 in Zanzibar, there was an increase in the number of clients tested from July to December 2020 compared to January to June 2020, with a pronounced increase in the number of clients tested positive from 0.6% of January to June 2020 to 0.7% of July to December 2020. The increased number of HIV testing clients and tested positive were due community outreach services of index testing and counselling services across the country. These results contrasted with those found in Japan, where providing COVID-19-related services became the primary burden at healthcare facilities as the number of tests completed in 2020 sharply decreased from 142,000 in 2019 to 69,000 in 2020 [8].

On the other hand, this report shows that the proportion of pregnant women who received antiretroviral therapy (ART) to lower the risk of mother-to-child transmission (91.7%), the proportion of infants born to HIV-positive mothers who had an HIV virological test within 2 months (87.6%), and the proportion of pregnant women who were aware of their positive HIV status (91.6%) all decreased between July and December 2020 in comparison to January-June 2020. Similar to this, the COVID-19 pandemic has hampered efforts in South Africa to limit mother-to-child HIV transmission (PMTCT). This remark was made during the interviews with 40 migrants, including 18 from within the country and 22 from overseas. This was brought on by border restrictions that hampered compliance and restricted their access to ARVs throughout the pandemic [9].

In comparison to January to June 2020, when key populations (MSM, FSW and PWID) were reached (6353), tested (5516), and found to be positive (120), fewer key populations were reached (2883), tested (2768), and identified as HIV-positive (73) between July and December 2020. An Indian study found that the initial shutdown had a significant impact on HIV testing for all typologies. The FSW, MSM, and Transgender numbers quickly bounced back after the unlock period [10].

The COVID-19 epidemic had a substantial effect on HIV care and treatment in Zanzibar. According to the institutions, there were 211 fewer new customers enrolled in April–June 2020 than in the other quarters of 2020, which had an effect on the number of clients receiving care and treatment (6797 and 6604). In order to determine how COVID-19 affects HIV care generally, Ellen et al. conducted a survey that identified 46 ART locations in Africa and the Asia–Pacific area. The COVID-19 epidemic caused some disruptions in the routine care and medical services at about 90% of the participating ART centres [11].

Zanzibar has put strict precautions in place to prevent the COVID 19 from spreading, like restricting hospital visitors. The provision of essential HIV services, such as care and treatment, services for key population, and PMTCT, was unintentionally hampered by these actions, despite the fact that they were necessary. Because they fear stigma associated with having to present their identity cards at the facility entrance main gate due to their undisclosed status, People Living with HIV (PLHIV) have difficulty accessing the care they need, including antiretroviral therapy (ART) and other important medications. Many hospitals and health facilities are working under partial capacity. Patients visiting these hospitals are needed to present a referral letter or proof that they are registered at a certain clinic. In this challenging time, the ARV 3-multi-month dispensing (3MMD) policy has been successfully implemented in all clinics for all stable clients who are 5 years of age or older, have received ART for at least 6 months, are free of adverse drug reactions that require regular monitoring, are not suffering with comorbidities and opportunity infections, have a good understanding of lifelong adherence of 95% and kept clinic visit appointments for the past 6 months, and are on first-line ARVs/second-line ARV with undetectable viral load (VL below 50 copies/ml). The vast majority of ART users in Zanzibar are represented by this group [7].

Additionally, a block system was implemented in Mnazi mmoja and Muembeladu CTCs to relieve client congestion and overcrowding. Clients were arranged in specific numbers, times, and dates for appointments to refill ARVs. Also, Fast Track Delivery of ARVs as Community Outreach Services to all stable clients through 3MMD was implemented at the Zanzibar People Living with HIV office (ZAPHA+), which is located at Welezo outside of Zanzibar town.

Campaigns to raise awareness of COVID-19 prevention and treatment have been run through a variety of media, including the Zanzibar Broadcasting Cooperation's (ZBC) television and radio, bronchures, and banners, in order to protect the general state of PLHIV in Zanzibar.

## Conclusion

The COVID-19 pandemic’s negative consequences on Zanzibar's HIV services bring to light the challenges that PLHIV and persons at risk of contracting HIV, particularly key population, confront in obtaining follow-up, care, and treatment services. The 3 Multi-Month Dispensing (3MMD) strategy for stable clients was crucial in addressing access issues, fostering trust among high-risk groups, and providing treatment for PLHIV throughout the epidemic. Facility block system ARV refills, community outreach services, including index testing, and community ARV refills at the ZAPHA + office for stable clients were prioritised as a novel strategy to guarantee the continued delivery of essential HIV services and can be used in the future in any situation.

## Data Availability

Data to be provided upon request.
